# Isolation of Protein and Fiber from Hot Pepper Seed Oil Byproduct To Enhance Rheology, Emulsion, and Oxidative Stability of Low-Fat Salad Dressing

**DOI:** 10.1021/acsomega.3c07410

**Published:** 2024-02-19

**Authors:** Esra Avci, Alican Akcicek, Zeynep Hazal Tekin Cakmak, Muhammed Zahid Kasapoglu, Osman Sagdic, Salih Karasu

**Affiliations:** † Department of Food Engineering, Faculty of Chemical and Metallurgical Engineering, Yildiz Technical University, Davutpasa Campus, 34210 Istanbul, Turkey; ‡ Bypro Functional Food and Biotechnology, Esenler, 34210 Istanbul, Turkey; § Faculty of Tourism, Department of Gastronomy and Culinary Arts, Kocaeli University, Kartepe, 41080 Kocaeli, Turkey; ∥ Istanbul Teknokent, Cerrahpasa Avcılar Campus, Istanbul University, 34320 Istanbul, Turkey

## Abstract

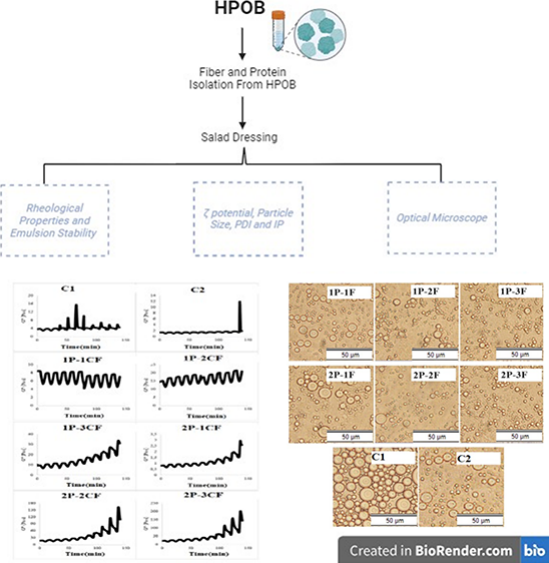

This research aimed to explore the potential utilization of protein (P) and fiber (F) extracted from cold-pressed hot pepper seed oil byproduct (HPOB) in the enhancement of the rheological properties, emulsion stability, and oxidative stability of a low-fat salad dressing with 10% oil content. The assessment involved the examination of several aspects, including the physical qualities such as emulsion stability, rheological behavior, and particle size as well as the microstructure and oxidative stability. It is worth mentioning that all emulsions had desirable characteristics, including shear-thinning behavior characterized by a consistency index ranging from 6.82 to 22.32 Pa s, as well as viscoelasticity and recoverability. These qualities were notably improved with the addition of P and F of HBOP. During the thermal stability testing, it was observed that the low-fat dressing containing 1% P–1F exhibited minor changes in the G* value, indicating its exceptional emulsion stability. The control salad dressings in C1 samples contained 30% oil. (B): C2: samples containing 10% oil (low-fat salad dressing sample) exhibited ζ-potential values of −34.70 and −46.70 mV. The samples 1P–1F and 2P–1F exhibited the highest ζ-potential values. Furthermore, the increase in F resulted in a reduction in droplet size and elicited elevated values for the induction period (IP), with the exception of samples containing 1% protein, 3% fiber, and 10% oil (1P–3F). The salad dressings that included P–F exhibited enhanced oxidative stability, demonstrated by their longer IP (ranging from 5.11 to 7.04 h) compared to the control samples. The formulation consisting of samples contained 1% protein, 1% fiber, and 10% oil (1P–1F) and samples contained 2% protein, 1% fiber, and 10% oil (2P–1F) exhibited superior ζ-potential, emulsion stability, and recovery rate compared to other formulations. The findings of this investigation indicate that the interaction of proteins and fibers extracted from HPOB exhibits the potential to enhance the rheological characteristics, emulsion stability, and oxidative stability of low-fat salad dressing.

## 1 Introduction

The issue of food waste and byproducts is widely acknowledged as a substantial global concern that poses a threat to the long-term sustainability of the food supply chain.^1^ The waste or byproducts have the potential to be transformed into valuable commodities such as polysaccharides, polyphenols, essential oils, dietary fiber, resins, taste compounds, and pigments, rather than being destroyed or thrown off in landfills.^1,2^

Peppers, regarded as beneficial vegetables, belong to the Solanaceae family under the Capsicum genus. *Capsicum annuum*, an indigenous species originating in the southern regions of North America, expanded its range to encompass Central and South America.^3^ Pepper cultivars that possess a high concentration of capsaicin, the chemical ingredient accountable for the distinctive pungency, are frequently denoted as hot peppers or chili peppers.^4^ Pepper is processed into diverse products like sauces, spices, and canned foods to satisfy market needs, resulting in substantial waste during processing, including stalks, unused flesh, and seeds.^5,6^ Notably, pepper seeds constitute a significant waste fraction containing protein, lipids, carbohydrates, minerals, vitamins, and bioactive compounds. Researchers are intrigued by the cold press extraction of pepper seed oil due to its aromatic richness and bioactive content.^6^ Chili seeds typically contain oil ranging from 20 to 25% w/w with more of their fatty acids being unsaturated.^7^ After the process of oil extraction, a residual meal that is low in fat but rich in protein, fiber, and carbohydrates is obtained. This meal possesses significant potential as a great resource for the development of innovative food products that are both nutritionally enriched and functionally beneficial.^4,8^ The cold-pressed hot pepper oil byproduct possesses a substantial amount of protein and fiber, making it a viable option as a source of alternative protein and fiber in many food products. The evaluation of this potential holds significance when considering both environmental and dietary aspects.

Salad dressing is a type of semisolid oil-in-water emulsion and functions as a condiment employed to elevate and transform the taste of salads and other foods.^9^ The process of salad dressing creation entails the combination of many components such as egg yolk, vinegar, oil, and spices. The fat percentage of this emulsion typically ranges from 30% to 80%, with variations governed by the specific classification of the dressing type.^10^ The high level of fat consumption led to several diseases such as diabetes, obesity, and cardiovascular disease.^11^ Reducing the amount of fat in salad dressing and mayonnaise is one of the customer requests. In food emulsions, fats have a variety of useful functions. They make highly specialized contributions to the taste, look, texture, and shelf life.^12^ Removing fat from dressings and mayonnaise can negatively impact the sensory and physicochemical properties. This poses challenges for manufacturers aiming to create novel, healthier products with plant-based ingredients and fewer calories. To maintain texture and taste, additives like texture enhancers or fat substitutes are essential, as consumers prioritize flavor and quality.^9^ The incorporation of fat replacers assists in mitigating the decline in product quality resulting from fat reduction. These replacers are classified based on their source as carbohydrate-based or protein-based.^13^ Protein particles utilized as fat replacers exhibit textural, rheological, and sensory characteristics comparable to fat. Polysaccharides act as fat replacers mainly by creating a gel-like matrix that stabilizes a significant amount of water, imparting creaminess and flow properties similar to fat.^14,15^

The objective of this study is to isolate protein and fibers from the residual byproduct of cold-pressed hot pepper seed oil, with the intention of generating additional value for this byproduct through the application of the extracted proteins in the food business. In order to achieve this objective, the protein and fibers derived from hot pepper seed oil byproduct (HPOB) were employed as substitutes for fat in a salad dressing sample. The aim was to investigate the impact of the interaction between protein and fiber on the stability of the emulsion as well as the rheological properties and microstructure characteristic of the low-fat salat dressing. A comparison was made with a sample of low-fat (C1) and full-fat salad dressing (C2).

## 2 Material and Methods

### 2.1 Materials

The cold-pressed HPOB used in the study was purchased from the ONEVA Foods Company in Esenyurt, Istanbul. The byproducts were ground in a lab mill (PX-MFC 90 D, Kinematica, Switzerland), sieved through the mesh (No. 140), and then kept at 10 °C in sealed polypropylene bags for additional examination. Sunflower oil, vinegar, and salt were obtained from a local market in Istanbul. Xanthan gum (XG) and egg yolk powder (EYP) were supplied by Sigma-Aldrich (Sigma Chemical Co., St. Louis, MO, USA).

### 2.2 Methods

#### 2.2.1 Chemical Composition of the HPOB

The oil content of the HPOB sample was determined using Soxhlet oil extraction with hexane, as per the AOAC 2003.05 standard procedure.^16^ For the moisture, ash, and protein contents of HPOB, standard AOAC method numbers 934.01, 942.05, and 990.03 were employed, respectively. The samples’ dry matter content was determined by drying them in an oven (FN 120, Nuve, Ankara, Turkey) at 105 °C for 4 h. The ash content was evaluated by burning the samples for 6 h at 550 °C.

#### 2.2.2 Fiber and Protein Isolation from HPOB

Fiber and protein isolation from HPOB was achieved through modification of their procedures.^17,18^ Base insoluble fiber was obtained after centrifugation of alkaline (pH 12) treatment, while base soluble fiber was isolated using ethanol washing and wet fractionation techniques, whereas protein was isolated using alkaline extraction/isoelectric precipitation techniques. First, a 1:15 (w/v) dilution of HPOB was adjusted to the pH 12 sample with 0.1 N NaOH. The highest soluble protein concentration was maintained at 60 °C throughout the preliminary experiments, whereby the samples were diluted at a ratio of 1:15. Ultrasound treatment was applied for a duration of 3 min, with a power output of 301.34 W. To remove the pellet, the liquid was centrifuged at 8942*g* for 15 min at 25 °C. After the centrifugation process, the base insoluble fiber was obtained. At a ratio of 1:1 (v:v), 95% ethanol was added to the resulting liquid fraction, and the mixture was centrifuged at 8942*g* for 15 min before precipitation. Base soluble fibers were isolated as a result of this technique. After centrifugation, the pH value at which the remaining liquid fraction’s ζ-potential value of 0 was determined. After the proteins’ isoelectric point was confirmed to be pH 5.5, they were precipitated by centrifugation at 8942*g* for 15 min. A freeze-drying system (Martin Christ GmbH, Beta 1–8 LSCplus, Germany) was used to lyophilize proteins. At this condition, the purity values of the protein and fiber were found as 96.25 and 80.84%, respectively. In this study, soluble fiber was used to prepare fiber-rich low-fat salad dressing samples (F), and protein was used to produce protein-rich salad dressing samples (P).

#### 2.2.3 Salad Dressing Preparation

The preparation of salad dressing samples was performed according to the previously described method.^19^ In the first step, XG (0.35%) was dissolved in water at 25 °C. HPOB fiber and protein were then added after the dispersion had been heated to 80 °C and stirred for 20 min. The mixture was refrigerated to 25 °C after salt was added. After the ingredients dissolved completely, the XG was thoroughly hydrated by being agitated in a magnetic mixer for 6 h at 1000 rpm. Homogenization was carried out using Ultra Turrax (Daihan, HG15D) at 10,000 rpm for 3 min following the addition of sunflower oil and EYP (3%). Eventually, salad dressing was obtained and pasteurized for 10 min at 65 °C. After pasteurization, salad dressing samples were cooled to 25 °C and analyzed. The sample of control salad dressings was made using the same methods. Sunflower oil was used in the formulation of the control samples (C1 and C2) at 10 and 30%, respectively. In all control samples, 0.35% of XG and 3% of EYP were present (Table 1). The pH value of the samples was ranged from 4.2 to 4.4. P–F samples were produced with 10% 0.35% of XG and 3% of EYP.

**Table 1:** Formulation of Salad Dressing Samples^a^

	water (%)	oil (%)	HPOB protein (%)	HPOB fiber (%)	vinegar (%)	EYP (%)	NaCl (%)	XG (%)
C1	58.65	30			7	3	1	0.35
C2	78.65	10			7	3	1	0.35
1P–1F	76.65	10	1	1	7	3	1	0.35
1P–2F	75.65	10	1	2	7	3	1	0.35
1P–3F	74.65	10	1	3	7	3	1	0.35
2P–1F	75.65	10	2	1	7	3	1	0.35
2P–2F	74.65	10	2	2	7	3	1	0.35
2P–3F	73.65	10	2	3	7	3	1	0.35

a EYP: Egg yolk powder, XG: xanthan gum, HPOB: hot pepper oil byproduct. C1: samples contained 30% oil. (B): C2: samples contained 10% oil (low-fat salad dressing sample), (1P–1F): samples contained 1% protein, 1% fiber, and 10% oil, (1P–2F): samples contained 1% protein, 2% fiber, and 10% oil, (1P–3F): samples contained 1% protein, 3% fiber, and 10% oil, (2P–1F): samples contained 2% protein, 1% fiber, and 10% oil, (2P–2F): samples contained 2% protein, 2% fiber, and 10% oil, (2P–3F): samples contained 2% protein, 3% fiber, and 10% oil. Different lowercase letters on the same line indicate significance between samples (*p* < 0.05).

#### 2.2.4 Rheological Analysis

To undertake rheological measurements (steady shear, dynamic rheological, and the three interval thixotrophy test (3-ITT)) of salad dressing mixes, a stress-controlled rheometer (MCR 302, Anton Paar, North Ryde, Australia) with a parallel-plate arrangement to shear and a Peltier heating/cooling system was utilized. The probe was PP50 (diameter 50 mm, Anton Paar, North Ryde, Australia) with a 0.5 mm gap. Each rheological measurement was conducted three times at 25 °C.

At shear rates ranging from 0.1 to 100 s^–1^, the steady shear rheological parameters of salad dressing blends were studied. After being fitted to the model parameters, steady shear rheological data were generated using the Power law model and nonlinear regression.
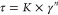
1where the τ value is the shear stress (Pa), *K* is the consistency coefficient (Pa s^*n*^), γ is the shear rate (s^–1^), and *n* is the flow behavior index.

The parallel plate system was used to investigate the dynamic rheology of salad dressing compositions. An amplitude sweep test at 0.1 and 100% strain was used to first detect the linear viscoelastic zone. Based on the results, the frequency sweep test was done at 0.1–10 Hz frequency range and 0.1–64 s^–1^ angular velocity. Based on their rotational velocity, the samples’ storage modulus (*G*′) and loss modulus (*G*″) were determined. The power law model and nonlinear regression were used to obtain the dynamic rheological parameters.
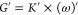
2
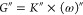
3where the *G*′ value is storage modulus (Pa), *G*″ value is loss modulus (Pa), ω is angular velocity value (s^–1^), *K*′, *K*″ are consistency coefficient values (Pa s^*n*^), and *n*′, *n*″ are flow behavior index values.

The 3-ITT rheological characteristics of salad dressing samples were determined to be 150 s^–1^ for a variable shear rate and 0.5 s^–1^ for a constant shear rate. When calculating the results, the linear viscoelastic range was taken into consideration. In the samples, this region ends at 50 s^–1^. During the first 100 s of the first interval, the salad dressing mixtures were subjected to a shear rate that was quite low (0.5 s^–1^). The determined shear rate was applied to 150 s^–1^ for 40 s during the second time interval. By subjecting the samples to low shear rates in the first time interval, the third time interval investigated the dynamic rheological behavior of the salad dressing mixes in the second period. The viscoelastic solid structure (*G*′) of the samples was altered. A second-order structural kinetic model was used to simulate how samples behaved throughout the third period.
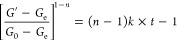
4where the *G*′ value denotes the change in the storage module (Pa); *G*′ denotes the initial storage module value (Pa) in the third time interval; *G*_e_ denotes the storage module (Pa) at the point at which the product has fully recovered, or when it is fully balanced; *k* denotes the thixotropic velocity constant and *t* → ∞ (nonstructured state) and *n* is assumed as 2 in the second order structural kinetic model.

#### 2.2.5 Emulsion Stability

The earliest description of the thermal loop test came from a previous study^20^ where the endurance of oil in water emulsions after 11 thermal cycles at high (from 23 to 45 °C) and low (from 5 to 23 °C) temperatures is assessed. A quick way to recreate temperature fluctuations in production, distribution, storage, and transport is to use a thermal loop test to detect structural changes. The emulsions were heated in cycles at various temperatures. The strain value was set to 0.5%, and the angular frequency was set to 10 Hz, respectively. The rates for cooling and heating were set at 11 K/min. The internal loop and rheometer software were used to find the maximum values for each cycle. The modification of modules from cycle to cycle reflects the morphological or structural alterations brought on by the temperature stress. The temperature loop test can therefore be used to determine the oil stability in water emulsions such as mayonnaise and salad dressing.

#### 2.2.6 Particle Size Distributions and ζ-Potential Values of Salad Dressing

A Malvern nanosizer (Nanosizer, Malvern Instruments, Worcester, UK) was used to measure the particle size distributions, average diameter (DZ), polydispersity index (PDI), and ζ-potential values in the salad dressing mixtures as described in Section 2.2.2.^19^

#### 2.2.7 Oxidative Stability

According to^21^ the description of the OXITEST Device (Velp Scientifica, Usmate, MB, Italy), the oxidative stability of the salad dressing samples was assessed. The OXITEST gadget was used to evaluate the samples for accelerated oxidation. Twenty grams of a sample was inserted into the device’s receptacle. The test for fast oxidation was carried out at 6 and 90 °C. When evaluating redox stability, the induction period number (IP) was taken into account.

#### 2.2.8 Light Microscope

A light microscope (Olympus BX41, Tokyo, Japan) was used to evaluate the morphology of the emulsions before and after the thermal loop test at 100× magnification. First, a coverslip was placed over a single drop of the sauce sample on a microscope glass. The stability of the emulsion was then thoroughly assessed by looking at several slide regions.

#### 2.2.9 Statistical Analyzes

Each replication received three parallel measurements, and each sample was manufactured in duplicate. The mean value and standard deviation of the data were displayed. The STATISTICA software program (Stat Soft Inc., Tulsa, UK) was utilized for statistical analysis. In Duncan’s analyses, the factor means were contrasted at a 0.05 confidence level. Using nonlinear regression analysis, power-law and second-order structural kinetic model parameters were created as a result of applied rheological research. The nonlinear regression analysis was carried out using STATISTICA software (Stat Soft Inc., Tulsa, UK).

## 3 Result and Discussion

The chemical compositions of HPOB were found as follows: moisture (%) 6.69 ± 0.03, protein (%) 20.25 ± 0.18, fat (%) 11.24 ± 0.13, carbohydrates (%) 57.72 ± 0.04, fiber (%) 31.91 ± 0.28, and ash (%) 3.83 ± 0.07, respectively.

### 3.1 Rheological Properties

#### 3.1.1 Steady Shear Rheological Properties

**Figure 1:**
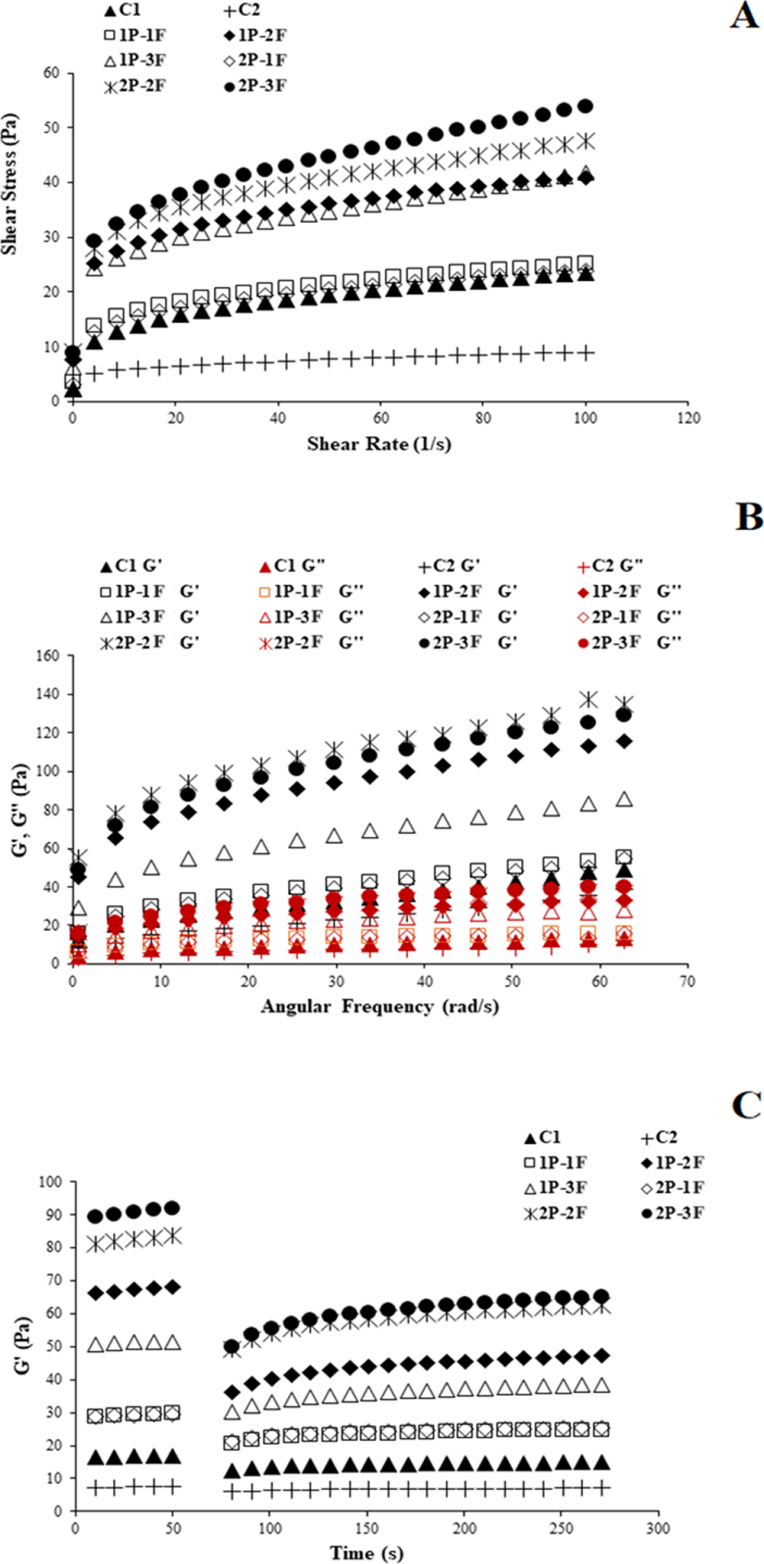
Rheological properties of salad dressing samples ((A): Steady shear rheological properties, (B): dynamic rheological properties, (C): 3-ITT rheological properties).

Figure 1a illustrates the flow curves for various salad dressing samples. All samples displayed shear-thinning behavior, indicating significant structural breakdown due to the deflocculating of oil droplets caused by shear forces.^22−25^ This behavior is typical for salad dressings and confirms their pseudoplastic flow behavior.^26^ Based on the data presented in the figure, it can be observed that samples containing both protein and fiber exhibit greater shear stress values when subjected to the same shear rate. This finding suggests that the viscosity values of samples stabilized with protein and fiber are greater. Despite having a lower oil content, these samples exhibit greater viscosity values compared to the C1 sample. This suggests that the protein–fiber interaction effectively regulates the continuous phase, leading to enhanced shear thinning behavior. A significant rise in viscosity was seen when the fiber content was raised from 1 to 2% while maintaining the same protein ratio. Nevertheless, when the fiber level was increased from 2 to 3%, there was only a moderate enhancement observed in the viscosity. No substantial increase in viscosity was detected when the protein level was raised from 1 to 2%. However, the simultaneous rise in protein and fiber contents resulted in a notable increase in viscosity. The observed phenomenon can be attributed to the interaction between the protein and fiber, which regulates the viscosity. Notably, 2P–3F exhibited the highest pseudoplasticity. Similarly,^27^ reported that the highest lime residue powder concentration in salad dressing showed the highest apparent viscosity with shear thinning behavior.

Based on Table 2, rheological measurements were performed on eight formulations, and their data were assessed using the power-law model. The model, well-fitted to the experimental data (*R*^2^: 0.970–0.992), enabled the determination of flow consistency index (*K*) and flow behavior index (*n*) values for each sample.

**Table 2:** Rheological Parameters of Salad Dressing^a^

	C1	C2	1P–1F	1P–2F	1P–3F	2P–1F	2P–2F	2P–3F
steady shear rheological parameters								
*K* (Pa s^*n*^)	6.82 ± 0.75^e^	3.64 ± 0.10^f^	9.75 ± 0.46^d^	19.36 ± 0.08^b^	16.50 ± 0.64^c^	9.26 ± 0.07^de^	22.32 ± 1.71^a^	20.34 ± 0.01^ab^
*n*	0.23 ± 0.04^a^	0.19 ± 0.00^ab^	0.20 ± 0.01^ab^	0.16 ± 0.00^b^	0.20 ± 0.01^ab^	0.21 ± 0.00^ab^	0.17 ± 0.01^b^	0.21 ± 0.00^ab^
*R*^2^	0.992	0.993	0.989	0.977	0.983	0.990	0.970	0.980
dynamic rheological parameters								
*K*′ (Pa s^*n*^)	10.16 ± 0.22^f^	4.95 ± 0.22^g^	15.50 ± 0.00^e^	45.42 ± 0.64^c^	28.56 ± 0.13^d^	14.23 ± 0.32^e^	55.18 ± 0.56^a^	49.89 ± 0.14^b^
*n*′	0.36 ± 0.01^b^	0.47 ± 0.01^a^	0.30 ± 0.00^c^	0.22 ± 00^e^	0.26 ± 0.00^d^	0.31 ± 0.01^c^	0.21 ± 0.00^e^	0.22 ± 0.00^e^
*R*^2^	0.977	0.983	0.994	0.997	0.993	0.992	0.990	0.996
*K*″ (Pa s^*n*^)	3.89 ± 0.02^e^	2.72 ± 0.07^f^	6.69 ± 0.04^d^	13.40 ± 0.08^b^	9.92 ± 0.06^c^	6.62 ± 0.23^d^	15.42 ± 0.39^a^	15.13 ± 0.21^a^
*n*″	0.28 ± 0.00^b^	0.32 ± 0.01^a^	0.21 ± 0.00^e^	0.22 ± 0.00^de^	0.24 ± 0.01^c^	0.19 ± 0.00^f^	0.20 ± 0.00^ef^	0.23 ± 0.00^cd^
*R*^2^	0.995	0.942	0.998	0.995	0.996	0.979	0.968	0.993
3-ITT rheological parameters								
*G*_0_^′^	16.23 ± 0.34^e^	6.19 ± 0.98^f^	19.22 ± 0.06^d^	34.15 ± 0.38^b^	28.36 ± 0.88^c^	20.21 ± 0.10^d^	45.34 ± 0.69^a^	46.17 ± 0.43^a^
*G*_e_^′^	22.11 ± 0.50^f^	8.07 ± 1.17^g^	25.54 ± 0.12^e^	50.90 ± 0.92^c^	41.76 ± 1.55^d^	26.57 ± 0.59^e^	65.97 ± 0.06^b^	69.30 ± 0.22^a^
*G*_e_^′^**/***G*_0_^′^	1.36 ± 0.00^bc^	1.31 ± 0.02^e^	1.33 ± 0.00^c^	1.49 ± 0.04^a^	1.47 ± 0.01^a^	1.32 ± 0.04^d^	1.46 ± 0.02^ab^	1.50 ± 0.02^a^
*k* × 1000	31.64 ± 3.48^ab^	34.17 ± 1.66^a^	27.42 ± 0.60^bc^	21.23 ± 0.32^d^	22.20 ± 0.28^cd^	27.94 ± 0.20^bc^	20.83 ± 1.32^d^	20.86 ± 0.20^d^
*R*^2^	0.996	0.998	0.997	0.998	0.995	0.996	0.997	0.998

a C1: samples contained 30% oil. (B): C2: samples contained 10% oil (low-fat salad dressing sample), (1P–1F): samples contained 1% protein, 1% fiber, and 10% oil, (1P–2F): samples contained 1% protein, 2% fiber, and 10% oil, (1P–3F): samples contained 1% protein, 3% fiber, and 10% oil, (2P–1F): samples contained 2% protein, 1% fiber, and 10% oil, (2P–2F): samples contained 2% protein, 2% fiber, and 10% oil, (2P–3F): samples contained 2% protein, 3% fiber, and 10% oil. Different lowercase letters on the same line indicate significance between samples (*p* < 0.05).

*K* values ranged from 6.82 to 22.32 Pa·s^*n*^, with *n* values varying between 0.16 and 0.23. These values differed across various salad dressing formulations. Moreover, in pursuit of a salad dressing characterized by elevated viscosity and an appealing mouthfeel, it becomes imperative that the thickening agents employed demonstrate a diminished flow index.^28,29^ Notably, C2 exhibited the lowest *K* value, while 2P–2F displayed the highest *K* value, highlighting the influence of the HPOB P–F content on *K* values.

Comparing all P–F of salad dressing samples with C1 and C2, all P–F samples showed higher *K* values despite having 66% less fat. In addition, interactions like P–F-XG play a significant role due to not only raised P–F’s *K* value alone but also when combined with XG. The hydrophilic interactions between P, F, and XG facilitate water molecule attachment, potentially leading to heightened *K* values.^30^ When the oil ratio decreased from 30 to 10%, it led to the *K* value of C1 decreasing from 6.82 to 3.85 Pa·s^*n*^. Although all P–F and C2 had the same amount of oil in the salad dressing, the increase in protein and fiber of byproduct caused the higher *K* value. The protein–polysaccharide interaction may be caused by an increase in the *K* value of salad dressing.^31^ These findings highlight the P–F compensatory role for the diminished oil content in influencing flow behavior. Additionally, *n* values below 1 indicate non-Newtonian properties in salad dressings, with decreasing *n* values corresponding to higher consistency coefficients. Pseudoplasticity, desirable for salad dressings, is characterized by *n* values approaching zero.^32^ found that the incorporation of HPOB significantly increased the *K* value of the low-fat control salad dressing due to the reduction in oil content’s impact on the structural integrity could be mitigated by HPOB addition. Due to the abundant fiber and protein content, the presence of water in the continuous phase remained stable, and the diminished viscosity and consistency were restored upon oil intake.^33^ The water-holding capacity of HPOB’s protein and fibers, along with interactions between protein and polysaccharides, likely restricted the movement of the continuous phase.^32^

#### 3.1.2 Dynamic Rheological Properties

The dynamic rheological properties of the eight samples of salad dressing are presented in Figure 1b. As the frequency was raised, there was an observed rise in both the magnitudes of the storage modulus (*G*′) and the loss modulus (*G*″). It is worth mentioning that all the samples exhibited viscoelastic properties, as indicated by the continuous dominance of the storage modulus (*G*′) over the loss modulus (*G*″), which suggests the formation of a gel-like structure resulting from a flocculated and interconnected network.^9^ The *G*′ value of C1 is greater than that of C2, indicating that emulsions with increasing oil content have proportionally increased *G*′ values.^34^ Reducing the oil content leads to a looser structure, resulting in decreased solid-like characteristics. Maintaining a solid-like structure is crucial for enhancing emulsion stability and product quality when reducing oil content.^33^

According to the data presented in Figure 1b, it can be observed that 2P–2F demonstrated the highest *G*′ values, which may be ascribed to the presence of covalent bonding and interactions followed closely by 2P–3F. The elucidation of this phenomenon is facilitated by the interplay between variables P and F, which serves to strengthen the gel-like structure of the samples of salad dressing. The fibrous polysaccharide generates a stable viscoelastic matrix inside the oil, effectively entrapping it within intermediate spaces. The higher oil retention capacity of food products contributes to the enhancement of their texture, taste, and processing characteristics.^35^

The viscoelastic characteristics of the salad dressing samples were assessed by utilization of the power-law model (Table 2). Based on the data shown in the table, it can be evident that the samples displayed a range of *K*′ and *K*″ values from 4.95 to 55.18 Pa s^*n*^ and 2.72 to 15.42 Pa s*^n^*, respectively. Additionally, the *n*′ and *n*″ values varied between 0.21 and 0.47, and 0.19 and 0.32, respectively (*R*^2^ = 0.977–0.997 and *R*^2^ = 0.942–0.998, respectively).

In all samples, the *K*′ value was higher than the *K*″ value, indicating a prevalence of elastic solid behavior over viscous behavior. 2P–2F exhibited the highest *K*′ and *K*″ values, while C2 had the lowest *K*′ and *K*″ values. In addition, despite the low fat content of the salad dressing, the solid-like character improved with the increase of P–F. A similar result was reported^19^ by the addition of flaxseed in a low-fat dressing. The presence of dietary fiber plays a significant role in altering the textural characteristics of salad dressing samples. It also helps prevent syneresis and contributes to the stabilization of high-fat content in the dressing.^36,37^ Also^38^ found that dietary fiber content affected the salad dressing qualities.

#### 3.1.3 Three-Interval Time Test

Salad dressings that exhibit poor recovery capabilities have a tendency to flow quickly over salads, a characteristic that is often not favored by customers. Hence, it is imperative for these items to demonstrate prompt recovery following sudden deformation.^9,39^Figure 1C illustrates the time-dependent alteration in the *G*′ value for the salad dressing samples. Figure 1C demonstrates how sample recovery following sudden deformation varies with the applied shear rate, reflecting differences in deformation magnitude. Notably, higher deformation corresponds to reduced self-recovery capacity across all samples.^30^

Table 2 presents 3-ITT parameters (*G*_0_, *G*_e_, *G*_e_/*G*_0_, *k* × 1000) acquired by using the second-order structural kinetic model. The observed ranges for *G*_0_, *G*_e_, *G*_e_/*G*_0_, *k* × 1000, and *R*^2^ were 6.19–46.17, 8.07–69.30, 1.31–1.50, 20.83–34.17, and >0.99, respectively. Notably, 1P–1F and 2P–1F displayed higher thixotropic behavior with the recorded values for *G*_e_/*G*_0_ and *k*.

The 3-ITT test is designed to evaluate the impact of sudden stresses on the rheological characteristics of food, simulating everyday movements such as shaking and pushing.^40^ The progression of *G*′ values for salad dressing samples is depicted in Figure 1C, illustrating the variance in recovery dependent on shear rate. The loss in sample recovery becomes more pronounced as the level of deformation increases. The data presented in Figure 1C illustrate the variations in *G*′ values as a function of time. It is observed that larger shear rates induce more structural deformation, resulting in a notable fall in *G*′. Consequently, regaining the original *G*′ values becomes challenging.

Table 2 shows 3-ITT data with the second-order kinetic model, offering *G*_0_′, *G*_e_′, and *k* values. *G*_e_′/*G*_0_′ ratio. The *G*_e_/*G*_0_ values found between 1.31 and 1.50, significantly affected the P–F of the byproduct when compared to the low-fat control salad dressing sample (*p* < 0.05). *G*_e_′/*G*_0_′ reflects the recovery degree and is positively influenced by P–F addition. Similar results were reported in our previous study for HPOB.^32^

Another parameter, the *k* value, indicates the recovery rate after sudden deformation, linked to the product structure. A higher *k* indicates a quicker recovery to the initial *G*′ level. *k* × 1000 ranged from 20.83 to 34.17 (Table 2), affected by product type and shear rate (*p* < 0.05). The inclusion of P–F effectively mitigated alterations in the rheological properties resulting from reduced oil content. This improved performance is attributed to increased intermolecular interactions facilitated by the formation of a protein–polysaccharide (small aggregate of hydrocolloid) network. The result showed that HPOB P–F could be used to improve the thixotrophy properties of low-fat salad dressing. Similar results were reported by refs (33,37,39) for chia seed oil byproduct, pumpkin seed oil byproduct, and flaxseed oil byproduct. 3-ITT suggests 1P–1F and 2P–1F enriched low-fat dressing can match full-fat dressing’s recovery, enhancing production possibilities.

### 3.2 Emulsion Stability

**Figure 2:**
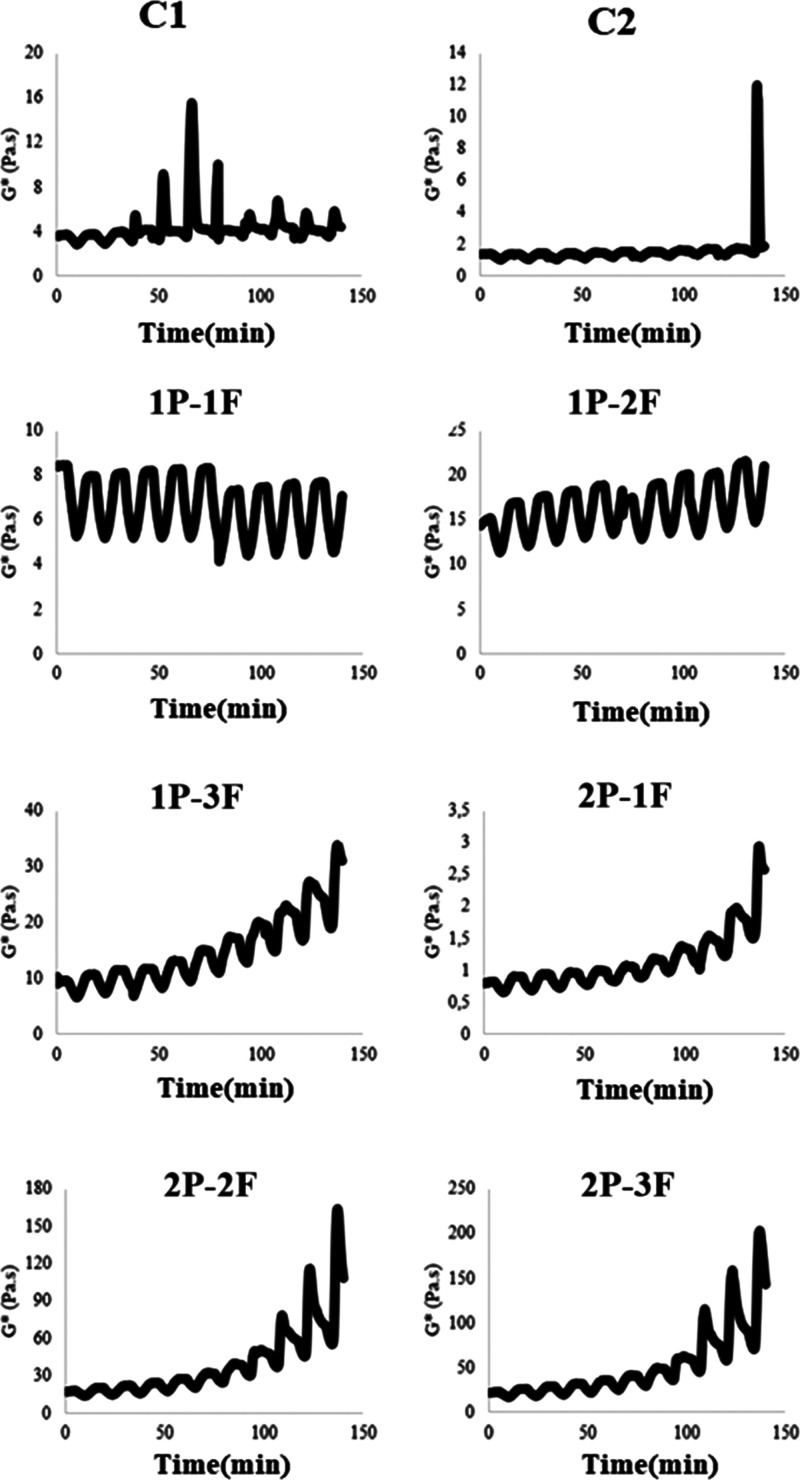
Emulsion stability test via the thermal loop test (change in G* values for samples: C1: samples contained 30% oil.: C2: samples contained 10% oil (low-fat salad dressing sample), (1P–1F): samples contained 1% protein, 1% fiber, and 10% oil, (1P–2F): samples contained 1% protein, 2% fiber, and 10% oil, (1P–3F): samples contained 1% protein, 3% fiber and 10% oil, (2P–1F): samples contained 2% protein, 1% fiber, and 10% oil, (2P–2F): samples contained 2% protein, 2% fiber, and 10% oil, (2P–3F): samples contained 2% protein, 3% fiber, and 10% oil.

The stability of emulsions is a crucial quality criterion for samples of salad dressing. The relevance of phase separation on the surface during storage in dressings that lack sufficient stability is addressed.^30^ Tekin, Avci, Karasu, and Toker^20^ conducted a study wherein emulsion stability was assessed through thermal loop tests, with higher shifts in G* indicating instability during thermal cycles. Figure 2 illustrates the variations in G* values observed during a series of loop tests conducted at elevated temperatures (ranging from 25 to 45 °C) for 10 iterations on samples of salad dressing. Significantly, the interaction between XG and P–F resulted in a strong framework, considerably augmenting the physical stability of low-fat salad dressings. Significant variations in G* shifts were noted between the C2 control sample and all 2P salad dressings, compared to the 1P salad dressing, during the high-temperature thermal loop test. The physical stability of the 1P–1F and 1P–2F samples was seen to be improved, as depicted in Figure 2.

High-temperature (25–45 °C) loop experiments, depicted in Figure 2, revealed G* changes over 10 loops. In these tests, G* decreased as the temperature rose. After the 10 cycles, significant G* change was seen in the low-fat C2 sample, denoting reduced emulsion stability. Sample 1P–1F displayed minimal G* change (Figure 2), indicating strong stability. However, 1P–3F, 2P–1F, 2P–2F, and 2P–3F exhibited notable G* increase between 25 and 45 °C due to various factors such as alterations in the solubility and viscosity of XG–P–F within the aqueous phase as well as shifts in viscosity within the dispersed phase, leading to instability processes.^32^

### 3.3 ζ-Potential, Particle Size Distribution, and IP Values of Salad Dressing Samples

The ζ-potential of salad dressings is a crucial indicator of their long-term stability. When the ζ-potential deviates from zero, it results in a positive or negative charge, reflecting stability.^41^ In Table 3, ζ-potential values for control and P–F samples ranged from −34.70 ± 0.26 to −46.70 ± 0.44 mV, demonstrating high ζ-potential values. Negative charge formation comes from bonding negative charges around oil droplets with polysaccharides via hydrophobic bonds. The control salad dressings, positioned within the ζ-potential values of salad dressings containing P–F (−34.70 and −46.70 mV), suggest potential long-term stability. The ζ-potential measurements of the 1P–1F and 2P–1F salad dressing samples exhibited greater values compared with the control samples. This observation suggests that the incorporation of P–F has the potential to enhance the stability of the emulsion (*p* = 0.05).

**Table 3:** ζ-Potential, Average Diameter, and IP Values of Salad Dressing Samples^a^

	ζ-potential (mV)	DZ (μm)	PDI	IP (h)
C1	–39.78 ± 0.12^b^	3.65 ± 0.22^ab^	0.95 ± 0.04^a^	3:33 ± 0.06^e^
C2	–36.52 ± 0.15^cd^	4.59 ± 0.18^a^	0.88 ± 0.12^ab^	2:51 ± 0.04^f^
1P–1F	–45.53 ± 0.25^a^	3.16 ± 0.47^abc^	0.60 ± 0.06^abc^	5:57 ± 0.04^c^
1P–2F	–34.70 ± 0.26^d^	0.91 ± 0.15^bc^	0.20 ± 0.09^c^	6:14 ± 0.05^b^
1P–3F	–38.10 ± 0.10^bc^	1.95 ± 0.42^abc^	0.37 ± 0.15^bc^	5:11 ± 0.10^d^
2P–1F	–46.70 ± 0.44^a^	2.43 ± 0.91^abc^	0.15 ± 0.07^c^	5:15 ± 0.07^d^
2P–2F	–36.33 ± 0.68^cd^	1.51 ± 0.88^bc^	0.58 ± 0.42^abc^	6:25 ± 0.11^b^
2P–3F	–37.20 ± 0.92^cd^	0.59 ± 0.07^c^	0.88 ± 0.12^ab^	7:04 ± 0.03^a^

a DZ: average diameter, PDI: polydispersity index, IP: induction period, C1: samples contained 30% oil. (B): C2: samples contained 10% oil (low-fat salad dressing sample), (1P–1F): samples contained 1% protein, 1% fiber, and 10% oil, (1P–2F): samples contained 1% protein, 2% fiber and 10% oil, (1P–3F): samples contained 1% protein, 3% fiber, and 10% oil, (2P–1F): samples contained 2% protein, 1% fiber, and 10% oil, (2P–2F): samples contained 2% protein, 2% fiber, and 10% oil, (2P–3F): samples contained 2% protein, 3% fiber, and 10% oil. Different lowercase letters on the same line indicate significance between samples (*p* < 0.05).

Particles possessing a potential greater than −30 mV have the ability to prevent aggregation via electrostatic interactions, hence resulting in the stability of emulsions.^35,42^ However, not all samples exhibited this capability; both the 1P–1F and 2P–1F samples efficiently inhibited aggregation by a combination of their potential and fiber shape.

Average diameter (DZ) significantly impacts the emulsion stability of salad dressing samples. The average diameter of the salad dressings ranged from 0.59 ± 0.07 to 4.59 ± 0.18 μm (Table 3). No statistically significant changes were detected, except in the 1P–2F, 2P–2F, and 2P–3F samples, when comparing them to the low-fat control sample and low-fat P–F salad dressing. The observed phenomenon indicated that a rise in F resulted in a reduction in droplet size, with the exception of the 1P–3F case. A marginal increase in droplet dimensions was noted for 1P–3F. The observed phenomena can be attributed to the depletion flocculation resulting from the presence of unadsorbed F in the continuous phase.^43,44^ However, PDI values of the salad dressing were found in the range of 0.15–0.95.

The oxidation stability of low-fat, which included HPOB P–F’s and control salad dressing samples, was assessed using the OXITEST equipment. Table 3 presents the IP. The salad dressing samples, when subjected to a temperature of 90 °C, exhibited IP values ranging from 2.51 to 7.04 h. The salad dressings that included P–F exhibited enhanced oxidative stability, as indicated by longer oxidation IP (5.11–7.04 h) compared to the C1 and C2 (3.33 and 2.51 h). The results of the salad dressing samples exhibited statistically significant variations with the exception of the 1P–3F and 2P–1F samples. The rise in P and F resulted in an increase in the IP value of salad dressing, with the exception of 1P–3F. Proteins have the capacity to inhibit lipid oxidation through ion chelation and the inherent antioxidant properties found in their amino acids,^45^ and the agricultural byproducts comprise noteworthy quantities of bioactive compounds, including dietary fiber containing associated phenolic compounds, referred to as antioxidant dietary fiber.^46^ The antioxidant effect of the P and F could play a major role in the increase in the IP value of the salad dressing sample. The introduction of additional F molecules, increasing the F concentration from 1P–2F to 1P–3F, resulted in droplet flocculation. This may be attributed to the presence of an excessive amount of unadsorbed F molecules in the aqueous phase of the emulsion.^47^ This result suggests that a concentration of 1% P was insufficient to completely cover the surfaces of the oil droplets. The observed insufficiency may be attributed to the decrease in polysaccharide content inside the limited interdroplet gaps, leading to the phenomena of flocculation and/or coalescence, rendering the system more vulnerable to oxidation.^48−50^ The results showed that P and F of the HPOB could be used to enhance the oxidative stability of the low-fat salad dressing.

### 3.4 Microstructure

**Figure 3:**
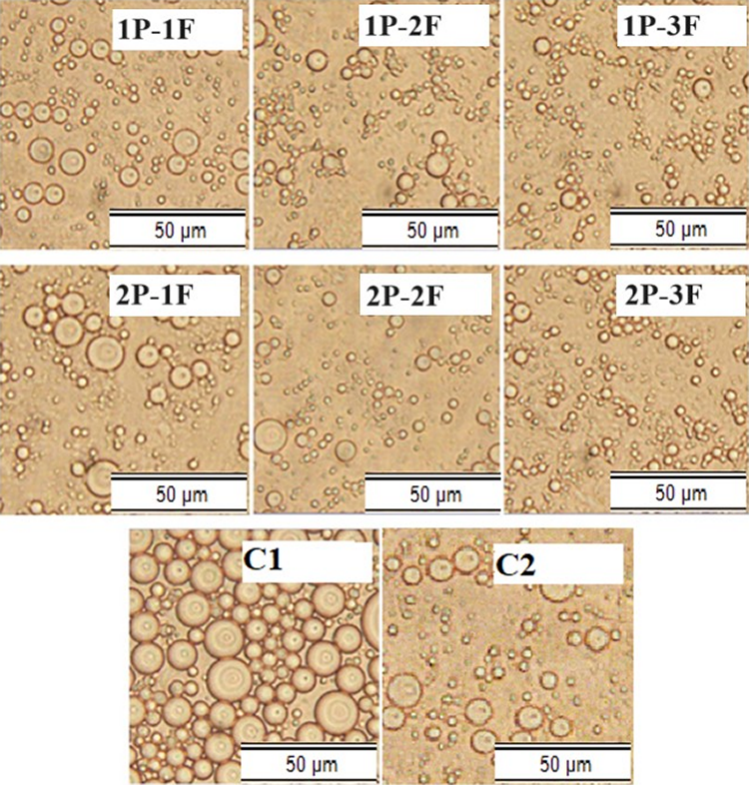
Optical microscope images C1: samples containing 30% oil. (B): C2: samples contained 10% oil (low-fat salad dressing sample), (1P–1F): samples contained 1% protein, 1% fiber, and 10% oil, (1P–2F): samples contained 1% protein, 2% fiber, and 10% oil, (1P–3F): samples contained 1% protein, 3% fiber, and 10% oil, (2P–1F): samples contained 2% protein, 1% fiber, and 10% oil, (2P–2F): samples contained 2% protein, 2% fiber, and 10% oil, (2P–3F): samples contained 2% protein, 3% fiber, and 10% oil.

The examination of salad dressing samples using a light microscope demonstrated that samples containing P–F exhibited smaller droplet sizes, although having a reduced oil concentration (Figure 3). Nevertheless, the concentration of F exhibited a negative correlation with particle size, with the exception of the 1P–3F sample. The results of this study are consistent with the analysis of emulsion stability and measurements of particle size, indicating that the presence of P–F impeded the coalescence of droplets by decreasing the mobility of the aqueous phase. The optical microscope pictures offer valuable insights into the flocculation process of the emulsion. The depletion flocculation process was found in the case of 1P–3F as a result of abundant unadsorbed polysaccharides present at the interface between oil and water.^49,50^ The combined data from emulsion stability, particle size measurements, and light microscope indicated P–F’s potential to enhance emulsion stability in reduced-fat salad dressings.

## 4 Conclusions

During the process of cold-pressing oils, byproducts that are abundant in bioactive chemicals, fiber, and protein are produced. The primary difficulty faced by producers of cold-pressed oil is the conversion of the resulting byproducts into resources of significant added value. This study aimed to explore the potential of HPOB protein and fiber in improving the rheological characteristics, emulsion stability, and oxidative stability of reduced-fat salad dressing. The addition of varying concentrations of PF led to notable improvements in the pseudoplasticity, viscoelasticity, and recoverable properties of low-fat salad dressings. Thermal loop testing, analysis of the ζ-potential, and recovery rate showed that 1P–1F enrichment contributed to enhanced emulsion stability. Furthermore, the IP values increased when the F concentration increased, except for the 1P–3F sample, due to the depletion flocculation. Based on these findings, when the low-fat control salad dressing is compared to P–F, it can be concluded that incorporating 1P–1F into low-fat salad dressing recipes could significantly enhance their rheological characteristics, oxidative stability, and emulsion stability.
